# Sex-specific cerebrovascular reactivity differences in autistic children related to functional connectivity

**DOI:** 10.1162/IMAG.a.1022

**Published:** 2025-11-17

**Authors:** Quimby N. Lee, Joshua K. Lee, Danielle J. Harvey, Peiying Liu, Christine Wu Nordahl, Audrey P. Fan

**Affiliations:** Department of Neurology, University of California Davis School of Medicine, Sacramento, CA, United States; MIND Institute, University of California Davis School of Medicine, Sacramento, CA, United States; Department of Psychiatry and Behavioral Sciences, University of California Davis School of Medicine, Sacramento, CA, United States; Department of Public Health Sciences, University of California Davis School of Medicine, Sacramento, CA, United States; Department of Diagnostic Radiology and Nuclear Medicine, University of Maryland School of Medicine, Baltimore, MD, United States; Department of Biomedical Engineering, University of California Davis, Davis, CA, United States

**Keywords:** autism spectrum disorder, cerebrovascular reactivity, resting-state functional MRI, functional connectivity, systemic physiology

## Abstract

Many studies utilize resting-state functional magnetic resonance imaging (rs-fMRI) metrics, such as functional connectivity (FC), to investigate the neuronal underpinnings of autism and identify functional brain networks related to autistic behaviors. However, fMRI indirectly measures neuronal activity by imaging local fluctuations in the blood oxygen level dependent (BOLD) signal, which, in turn, rely on the cerebrovascular system to efficiently direct oxygenated blood flow. Most rs-fMRI studies of autism interpret group differences in FC as autism-related changes in neuronal activity, without considering the underlying vascular function. Yet, atypical cerebrovasculature has been identified in preclinical and post-mortem studies of autism, strongly underscoring the need to characterize cerebrovascular differences to enhance our neurobiological understanding of autism. We evaluated relative cerebrovascular reactivity (rCVR) in autistic and non-autistic children using a novel measure of local brain vasodilatory capacity based on rs-fMRI. We leveraged the cross-sectional Autism Brain Imaging Data Exchange repository to quantify rCVR in 199 non-autistic (74 female) and 95 autistic (16 female) children, 9–12 years old. We identified sex-specific differences in rCVR in autism, particularly in right-frontal brain regions, where rCVR was increased in autistic females compared to non-autistic females. Then, within the same rs-fMRI scans, we demonstrated that rCVR in the right inferior frontal gyrus was positively associated with its FC to regions associated with attentional control in girls, suggesting that cerebrovascular differences may differentially affect FC findings between regions and sexes in children. Our study highlights potential sex differences in cerebrovascular function in autism that enhance our neurobiological understanding of autism and improve interpretations of rs-fMRI findings in children.

## 1 Introduction

Autism spectrum disorder (ASD or autism) is a heterogenous neurodevelopmental condition characterized by social challenges and restrictive and repetitive behaviors that is associated with multiple medical and psychiatric comorbidities (Mannion & Leader, 2013; Simonoff et al., 2008; Weir et al., 2021). A large literature has leveraged resting-state functional magnetic resonance imaging (rs-fMRI) to investigate the neurobiology of autism based on the blood oxygen level dependent (BOLD) signal, which reflects differences in blood oxygenation in response to neuronal activity. Rs-fMRI studies use a variety of metrics, including the temporal correlation in regional BOLD signals (functional connectivity; FC) to infer networks in the brain and how they differ in autism (Hull et al., 2016). However, the current autism rs-fMRI literature is inconsistent with mixed reports of increased and decreased brain connectivity across multiple resting-state neuronal networks (Abbott et al., 2016; Nomi & Uddin, 2015) and sex differences in these effects (Lawrence et al., 2020; J. K. Lee et al., 2020; Li et al., 2023). In general, these discrepancies have been attributed to the heterogeneity of autism phenotypes and their associated neuronal differences.

**Fig. 1.**
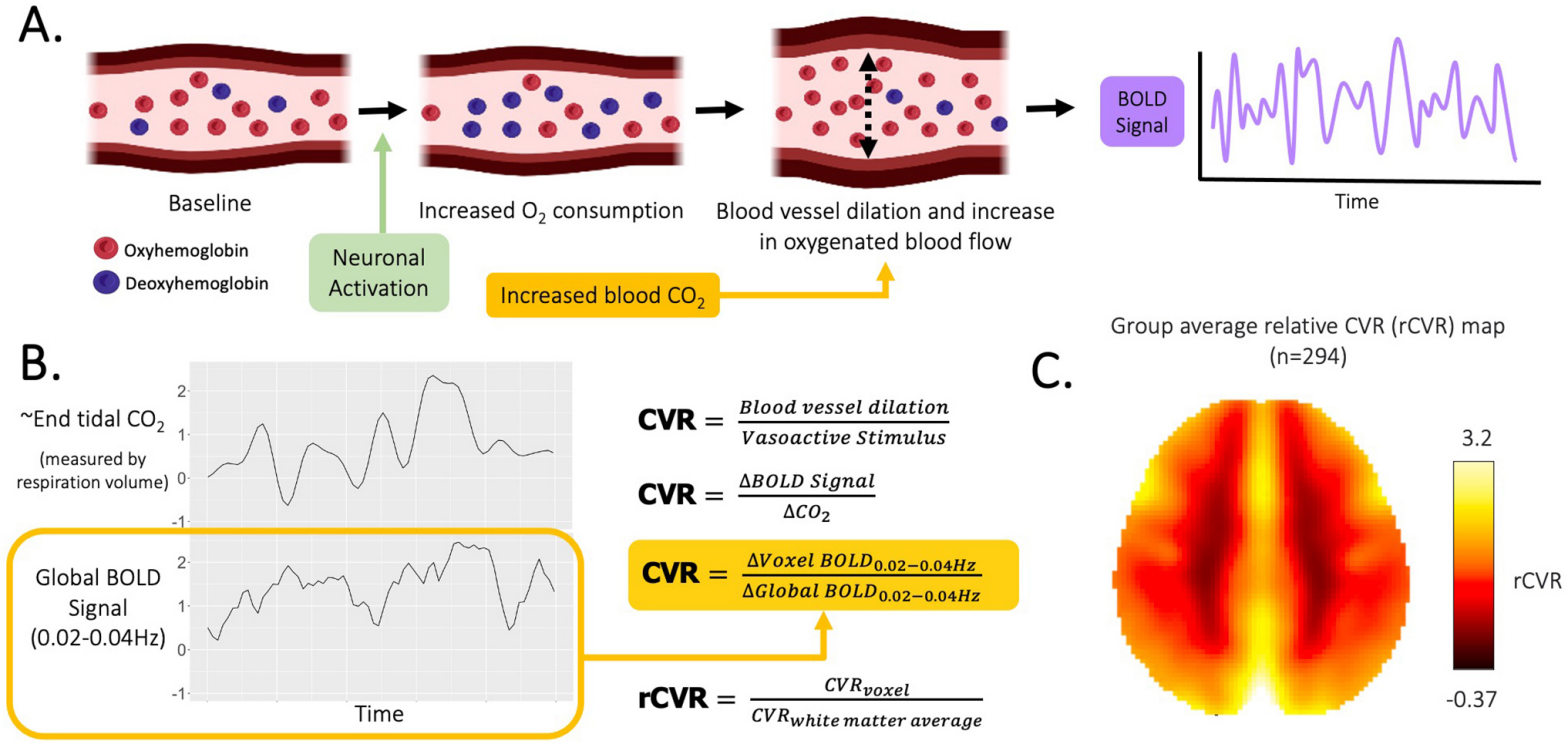
Relative cerebrovascular reactivity (rCVR) mapping using resting-state functional MRI without gas challenges. (A) The blood oxygen level dependent (BOLD) signal of functional MRI indirectly measures neuronal activity by detecting changes in oxygenated blood flow, which occurs through local cerebral blood vessel dilation. In addition to neuronal activity, changes in blood CO_2_, a vasodilator, can induce changes in local oxygenated blood flow, and thus the local BOLD signal. (B) Changes in CO_2_ during natural breathing are reflected in the global BOLD signal at 0.02–0.04 Hz, the frequency of respiration volume changes at rest. Thus, we can calculate regional CVR, or blood vessel dilation per vasoactive stimulus, as the change in local BOLD signal per change in global BOLD signal at 0.02–0.04 Hz. The representative end-tidal CO_2_ and global BOLD signal timeseries were generated from data from a different study. (C) The group average rCVR map highlights characteristic gray-white matter contrast, as gray matter is more highly vascularized than white matter.

Yet in addition to neuronal activity, the BOLD signal also reflects vascular changes that drive oxygenated blood flow. These vascular factors, such as local brain vascular capacity and cardiac and respiratory dynamics, are known to affect the BOLD signal within a brain region and BOLD correlations between regions (Fig. 1A) (Birn et al., 2006; Chang et al., 2013; Chang & Glover, 2009; Golestani et al., 2016). Despite the extensive use of rs-fMRI in autism research, the extent to which heterogeneity in cerebrovascular differences may contribute to disparate rs-fMRI findings, and whether this may differ regionally or between sexes, has not been examined. Preclinical and postmortem studies have identified cerebrovascular differences in some autistic individuals (Azmitia et al., 2016; Fiorentino et al., 2016; Jiménez et al., 2022; Ouellette et al., 2020), but no in-vivo human studies have evaluated these potential differences and how they may impact our understanding of autism as assessed through rs-fMRI. Characterizing cerebrovascular differences in autism is, thus, critical to disentangling neuronal versus vascular interpretations of rs-fMRI findings and informing new biomarkers to understand the etiology of autism.

Most rs-fMRI studies in autism focus on neuronal dynamics by removing systemic physiology as a confound during preprocessing (e.g., regressing out global BOLD signals). However, regional responses to systemic physiology may vary between autistic and non-autistic individuals and implicate underlying cerebrovascular differences. Indeed, preclinical studies have identified differences in brain angiogenesis (i.e., blood vessel formation) and downregulation of vascular-endothelium associated genes in mouse models of autism (Jiménez et al., 2022; Ouellette et al., 2020). Post-mortem studies have found different patterns of brain angiogenesis and blood-brain-barrier integrity in autistic brains (Azmitia et al., 2016; Fiorentino et al., 2016). Cerebrovasculature is integral to supporting the brain’s high metabolic demand, which comprises 20% of total cardiac output (Attwell & Laughlin, 2001; Xing et al., 2016). During neurodevelopment, the brain’s energy consumption is doubled, further emphasizing the importance of cerebrovascular efficiency during this critical period (Chugani, 1998; Kuzawa et al., 2014). Alterations to nutrient delivery through cerebral blood flow could affect cortical development and ultimately increase susceptibility to neurodevelopmental conditions (Iadecola, 2017; Penna et al., 2021). Recent epidemiological and genetic studies have indicated an increased risk of cardiovascular conditions (e.g., hypertension, dyslipidemia, and atherosclerotic macrovascular disease) (Dhanasekara et al., 2023) and stroke (Jin et al., 2024) in autistic individuals; however, how these vascular comorbidities may manifest in the brain is unknown. In this study, we evaluate cerebrovascular reactivity (CVR), a novel measure of cerebrovascular function derived from rs-fMRI scans, to investigate cerebrovascular differences across the brain in autistic compared to non-autistic children in-vivo, and how CVR contributes to FC in these populations.

CVR, or the capacity for blood vessels to dilate in response to a vasoactive stimulus, is a key indicator of cerebrovascular health. Previous studies have administered vasodilatory stimuli (e.g., breathing air with increased CO_2_) to participants during fMRI to measure CVR as the percent change in BOLD signal per unit change in vasodilatory stimulus (e.g., end-tidal CO_2_) in adults (Liu et al., 2019). This technique is widely used to study cerebrovascular conditions, such as stroke and arterial stenosis (Donahue et al., 2013; Geranmayeh et al., 2015). In healthy aging studies, lower CVR in frontal and temporal areas has been associated with cognitive decline (Catchlove et al., 2018; Peng et al., 2018). However, this method of CVR assessment is inaccessible to pediatric and autistic populations due to the challenges in administering and recording vasoactive stimuli in children. Previous CVR studies have utilized breath-hold paradigms during fMRI to innately increase arterial CO_2_ in children (Zvolanek et al., 2022) and identified network-specific changes in CVR during development (D. Y. Chen, Di, & Biswal, 2024). However, these studies require additional respiratory monitoring apparatus, are limited by sample size, and are restricted to children who can adhere to the breath-hold tasks.

To circumvent these challenges, recent studies have proposed using the global BOLD signal at respiratory frequencies to estimate arterial CO_2_ during natural breathing and calculate relative CVR (rCVR) from standard resting-state fMRI scans acquired without additional equipment or instructions (Fig. 1B) (Liu et al., 2017, 2021). This new method of estimating rCVR has been used to capture treatment effects of revascularization surgery in patients with Moyamoya disease (Liu et al., 2021) and identify positive correlations between rCVR in left-frontal brain regions to general cognition, executive function, and information processing speed in healthy aging (Ni et al., 2021). With this innovation, rCVR can be investigated using standard rs-fMRI scans, which increases the utility of large rs-fMRI repositories and enables parallel analyses of rCVR and other rs-fMRI derived metrics (e.g., FC) within a single scan to increase biological consistency. For the first time, we leverage this resting-state method in a large, cross-sectional pediatric cohort, the Autism Brain Imaging Data Exchange (ABIDE) database, to investigate regional rCVR differences in autism compared to typical development and how they are related to FC in children.

## 2 Methods

### 2.1 Participants

We utilized data from ABIDE, a multi-site repository of previously collected structural and rs-fMRI scans from autistic and non-autistic individuals (Di Martino et al., 2014, 2017). To improve data harmonization, eight acquisition sites with similar rs-fMRI parameters (repetition time[TR] = 2-3 s, echo time[TE] = 20-30 ms) were considered in this study. Site characteristics are provided in Supplementary Table 1. Our participant inclusion criteria were (i) children ages 9–12 years old, (ii) participants with fewer than 25% of rs-fMRI frames censored for excess motion (framewise displacement [FD]>0.25 mm) and at least 4 minutes of rs-fMRI data after frame censoring, and (iii) images with no major imaging artifacts or distortion upon visual inspection. To help isolate diagnostic differences from age-related effects, an age range (9–12 years old) with relatively stable metabolic (Kuzawa et al., 2014) and neurovascular coupling changes (Baller et al., 2022) in non-autistic development was selected. Children in the autistic group were diagnosed with ASD using the Autism Diagnostic Interview-Revised (Lord et al., 1994) and Autism Diagnostic Observation Schedule (ADOS)-Generic or ADOS-2 (Lord et al., 2012) by a clinician trained to research standards. Non-autistic children reported no history of neurological or psychiatric conditions. Details on site-specific inclusion criteria and informed consent are provided on the ABIDE website (https://fcon_1000.projects.nitrc.org/indi/abide/). Out of 389 total participants across eight sites, 199 non-autistic (74 female) and 95 autistic (16 female) children met all inclusion criteria and were included in this study. Participant characteristics are provided in Table 1. Age did not significantly differ between diagnostic or sex groups. IQ was significantly lower in autistic males and autistic females compared to their non-autistic counterparts. IQ did not significantly differ between sexes in autistic or non-autistic groups.

**Table 1.** Participant characteristics.

	Autistic (N = 95)		Non-autistic (N = 199)	
	Males	Females	Males	Females
Total number of participants	79	16	125	74
Age	10.6 (0.9)	10.7 (1.0)	10.5 (0.9)	10.3 (0.8)
Full scale IQ*	107.1 (19.4)	104.4 (10.5)	113.7 (12.1)	116.1 (10.8)
Autism diagnostic interview – revised (ADI-R)				
ADI-R number of participants	79	15	-	-
ADI-R social	20.0 (5.0)	16.7 (6.0)	-	-
ADI-R verbal	16.3 (4.7)	13.3 (4.8)	-	-
ADI-restrictive and repetitive behaviors	6.2 (2.4)	6.1 (2.7)	-	-
Autism diagnostic observation schedule – 2 (ADOS-2)				
ADOS-2 number of participants	55	12	-	-
ADOS-2 total	12.1 (4.1)	11.3 (3.7)	-	-
ADOS-2 calibrated severity score	7.0 (1.8)	6.5 (2.0)	-	-
Autism diagnostic observation schedule – generic (ADOS-G)				
ADOS-G number of participants	24	4	-	-
ADOS-G total	11.8 (4.4)	12.8 (3.6)	-	-

Data are summarized as group mean (standard deviation).

* Significant difference between autistic and non-autistic participants (p *< *0.05).

### 2.2 Imaging analyses

#### 2.2.1 Preprocessing

Structural images were brain extracted and segmented into gray matter, white matter, and cerebrospinal fluid using the FSL brain extraction tool (BET) and FMRIB automated segmentation tool (FAST), respectively (Smith, 2002; Zhang et al., 2001). Resting-state fMRI scans were motion corrected and 18 motion parameters (6 directions, temporal derivative, and square of motion), a linear trend over time, and imaging frames with FD greater than 0.25 mm were censored using linear regression (Jenkinson et al., 2002). Resting-state fMRI images were then spatially smoothed (Gaussian kernel full-width-half-max = 8 mm).

#### 2.2.2 CVR

The average whole-brain BOLD signal reflects respiratory dynamics and systemic changes in arterial CO_2_, especially at the frequency of respiratory volume changes during natural breathing, 0.02–0.04 Hz (Fig. 1B) (Birn et al., 2006; Liu et al., 2017). We used this frequency-filtered whole-brain BOLD signal as our surrogate timeseries for arterial CO_2_ changes. To calculate the voxel-wise change in BOLD signal per change in arterial CO_2_ (i.e., CVR), we regressed each voxel’s BOLD signal on the frequency-filtered whole-brain BOLD signal (Fig. 1B). Voxels with inverse temporal signal-to-noise ratio greater than the 98^th^ percentile of voxels were excluded from analysis to remove unwanted signals from large vessels, cerebrospinal fluid, or image distortion. Resulting β maps from linear regression were normalized to averaged β across white matter voxels to produce rCVR maps. White matter has 70–75% less vasculature and significantly lower CVR compared to gray matter (Lu et al., 2005; Thomas et al., 2014) and thus provides a suitable region for within subject normalization that is expected to have smaller CVR changes compared to gray matter between groups. Importantly, white matter β did not significantly differ between diagnostic groups. As a supplemental analysis, we compared rCVR results using global normalization instead of white matter normalization and there were no differences in the overall pattern of results (Supplementary Fig. 1). Voxel-wise CVR calculation was performed in native fMRI space using MATLAB scripts adapted from the seeVR toolbox (Bhogal, 2021). CVR maps were then warped to the T1-anatomical image and Montreal Neurological Institute (MNI) standard space for group-level comparison, using FMRIB linear and nonlinear registration tool (FLIRT/FNIRT) respectively (Jenkinson et al., 2002; Jenkinson & Smith, 2001).

**Fig. 2.**
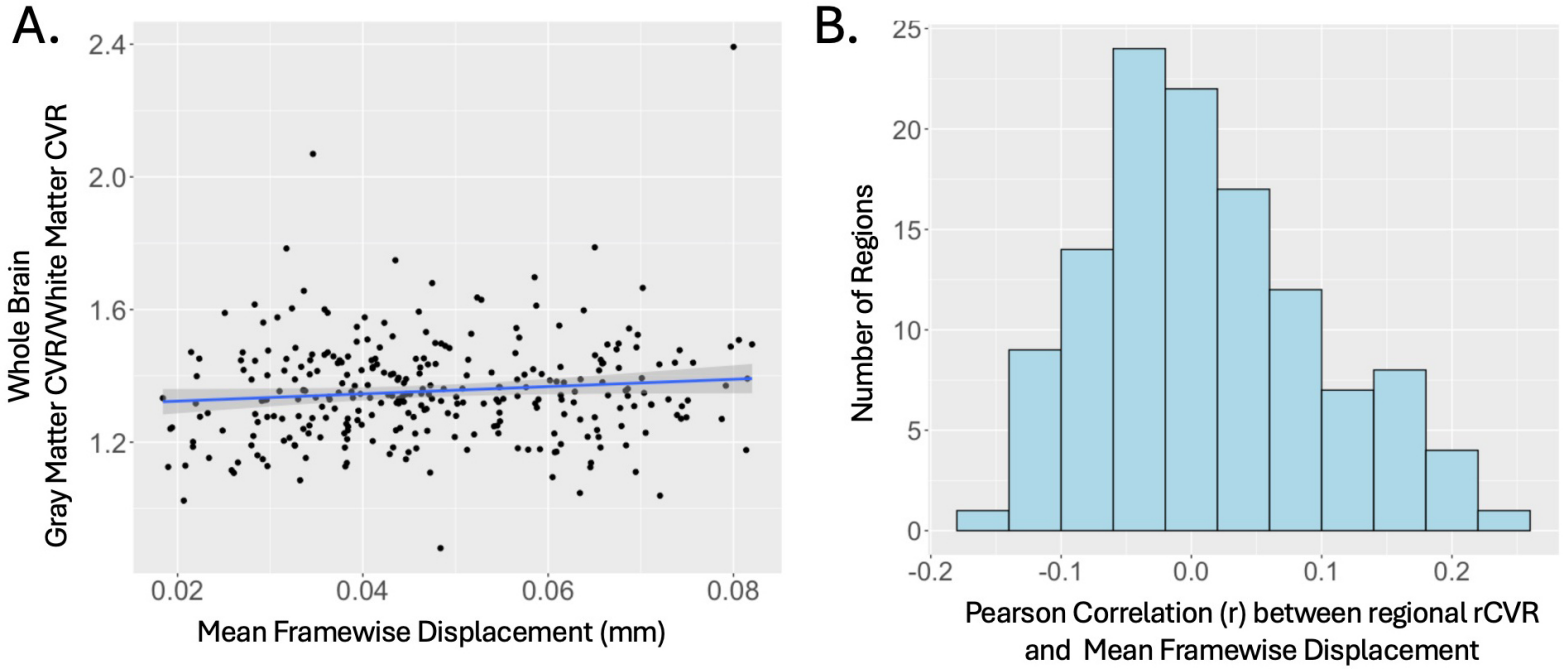
Relative cerebrovascular reactivity (rCVR) is not significantly associated with motion. (A) Across participants, whole-brain gray matter was not significantly related to motion, as measured by mean framewise displacement (mFD). (B) Across regions, regional rCVR were not significantly correlated with mFD.

rCVR is a within-subject relative value that is robust to motion. After within-subject normalization, average gray matter rCVR was not associated with participant motion, as measured by mean FD (Fig. 2). Similarly, regional rCVR for individual gray matter regions did not show significant correlations with mean FD.

#### 2.2.3 Functional connectivity

In addition to the preprocessing steps listed above, for FC analyses, white matter and cerebrospinal fluid timeseries were included as nuisance regressors in linear regression and data were frequency filtered from 0.008 to 0.1 Hz. Resting-state images were transformed to T1-anatomical and MNI space before FC calculation. Using AFNI software (Taylor & Saad, 2013), we measured region-to-region FC as the Fisher z-transformed Pearson correlation between the BOLD signal of the seed region (described below) and each gray matter region across the brain.

### 2.3 Data harmonization

To mitigate the effects of different scan acquisitions on our analyses, we utilized ComBat (Johnson et al., 2007), a data-driven location (mean) and scale (variance) adjustment based on empirical Bayes estimation that has previously been used to harmonize MRI metrics across scanners and acquisition sites (Fortin et al., 2017, 2018). In this study, we harmonized summary MRI measures of rCVR and FC in separate ComBat models. In each model, features represented regional gray matter averages. Diagnosis, sex, and mean-centered age were included as biological covariates of interest. Distributions of rCVR and FC values for each site before and after data harmonization are provided in Supplementary Figures 2-3. Harmonized data were then used for all subsequent statistical analyses.

### 2.4 Statistical analyses

For each participant, rCVR was calculated voxel-wise and then averaged within 91 gray matter regions defined using the Harvard-Oxford atlas (Desikan et al., 2006; Frazier et al., 2005; Goldstein et al., 2007; Makris et al., 2006). General linear models in R were used to compare rCVR between diagnostic and sex groups for each region. Covariates included diagnosis (autistic or non-autistic), sex, the diagnosis-by-sex interaction, mean-centered age, and mean FD. Sex was coded as -1 and 1 for male and female respectively to measure an overall diagnostic effect across sex. Diagnosis was coded as -1 and 1 for autistic and non-autistic participants respectively. In regions with significant diagnosis-by-sex effects, post-hoc linear models were performed within sex subgroups to disentangle sex-specific diagnostic differences. Since we conducted exploratory analyses across a large atlas (91 regions) with a novel metric, we provide a comprehensive characterization and report uncorrected p-values.

We then evaluated how these differences in regional rCVR may impact FC to other regions across the brain. Each region with diagnostic or sex differences was used as a seed for FC analysis. We then measured the association between rCVR in each seed region to its FC to all other gray matter regions in the Harvard-Oxford atlas. General linear models investigating the association between rCVR and FC were conducted within sex and diagnosis groups, with sex or diagnosis, mean-centered age, and mean FD as covariates. False discovery rate (FDR) adjustment was used to correct for multiple comparisons in FC analyses.

## 3 Results

### 3.1 Diagnosis and sex effects on rCVR

**Fig. 3.**
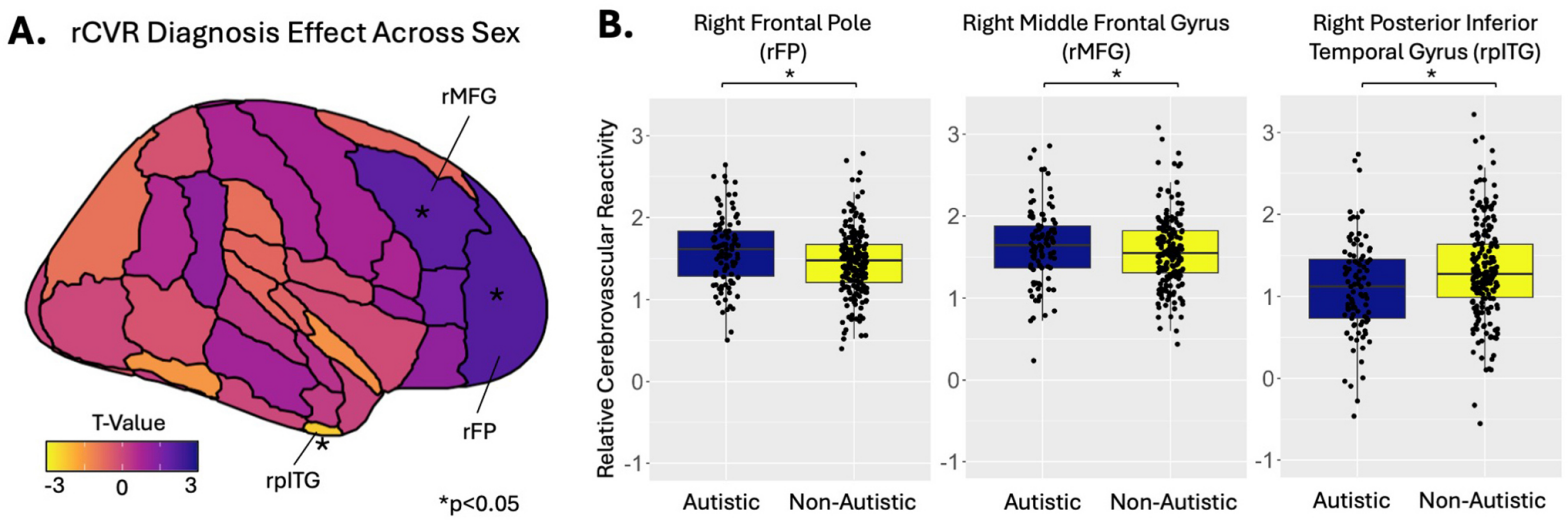
(A) Diagnostic effects on relative cerebrovascular reactivity (rCVR) across sex. (B) Autistic children had higher rCVR in the right frontal pole (rFP) and right middle frontal gyrus (rMFG), but lower rCVR in the right, posterior inferior temporal gyrus (rpITG) compared to non-autistic children. *p *< *0.05.

Across sex, autistic children had higher rCVR in the right frontal pole (rFP; *β* = 0.07, p = 0.03) and right middle frontal gyrus (rMFG; *β* = 0.07, p = 0.04), but lower rCVR in the right, posterior inferior temporal gyrus (rpITG; *β* = -0.11, p = 0.02) compared to non-autistic children (Fig. 3; Table 2). Across diagnosis, females had lower rCVR in the subcallosal cortex (*β* = 0.1, p = 0.02), precuneus cortex (*β* = 0.08, p = 0.04), and left posterior parahippocampal gyrus (lPHC; *β* = 0.07, p = 0.04), but higher rCVR in the left planum polare (*β* = 0.09, p = 0.02) compared to males (Table 2).

**Table 2.** General linear model of relative cerebrovascular reactivity.

Effect	Region	Beta	p-value	Relation
Dx × Sex	Right inferior frontal gyrus	0.13	0.003	ASD: F>MNA: F<M Females: ASD>NA Males: ASD~NA
	Right orbitofrontal cortex	0.08	0.01	ASD: F~MNA: F<M Females: ASD>NA Males: ASD~NA
Dx	Right frontal pole	0.07	0.03	ASD>NA
	Right middle frontal gyrus	0.07	0.04	ASD>NA
	Right posterior inferior temporal gyrus	0.11	0.02	ASD<NA
Sex	Subcallosal cortex	0.1	0.02	F<M
	Precuneus cortex	0.08	0.04	F<M
	Left posterior parahippocampal gyrus	0.07	0.04	F<M
	Left planum polare	0.09	0.02	F>M

Diagnosis (Dx); autism spectrum disorder (ASD); non-autistic (NA); female (F), male (M), no significant difference (~).

### 3.2 Diagnosis-by-sex effect on rCVR

**Fig. 4.**
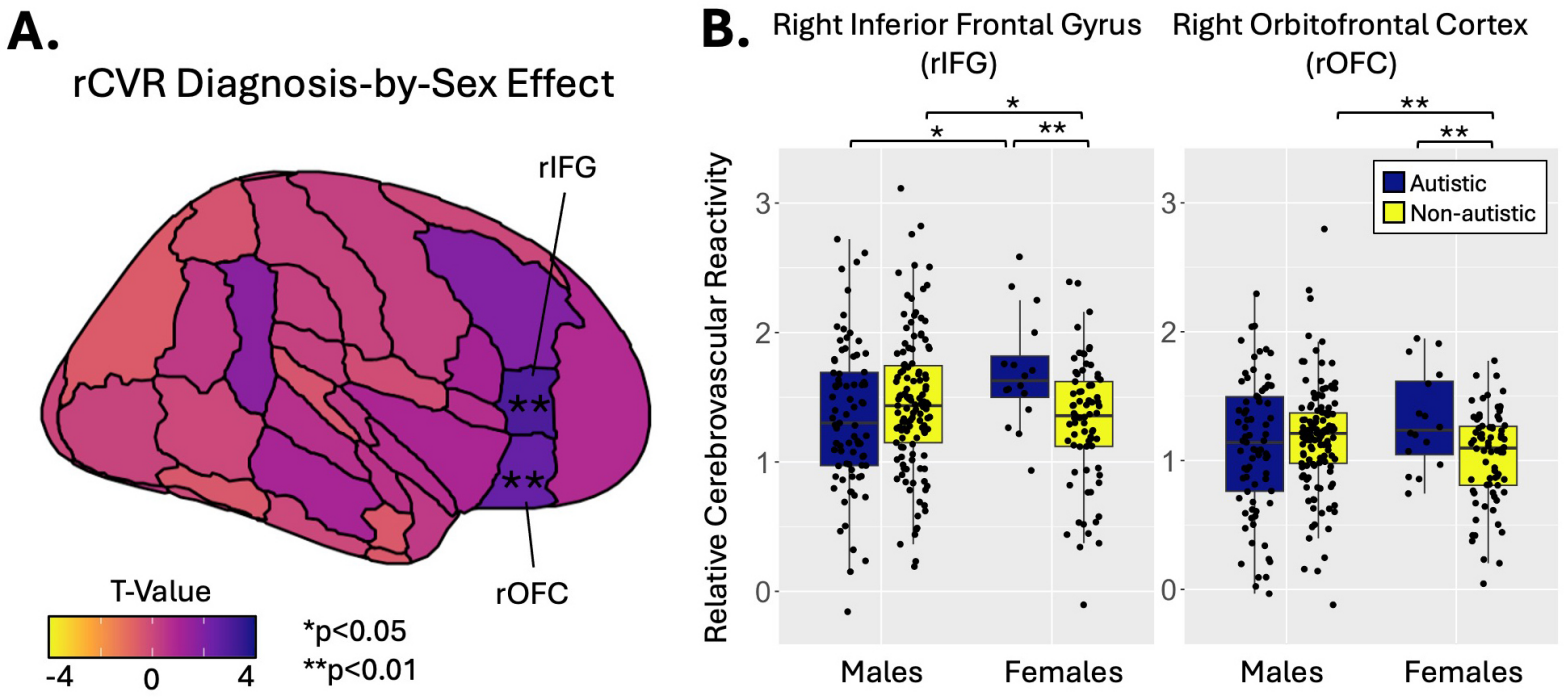
Diagnosis-by-sex effects on relative cerebrovascular reactivity (rCVR). (A) Significant diagnosis-by-sex effects were identified in the right inferior frontal gyrus (rIFG) and right orbitofrontal cortex (rOFC). (B) In the rIFG and rOFC, rCVR was higher in the autistic group compared to the non-autistic group in females but not males. In the non-autistic group, females had lower rCVR than males in both regions. However, in the autistic group, females had higher rCVR than males in the rIFG. *p *< *0.05, **p *< *0.01.

Within the same general linear model, diagnosis-by-sex interactions were identified in the right inferior frontal gyrus (rIFG; p = 0.003) and right orbitofrontal cortex (rOFC; p = 0.01; Fig. 4; Table 2). In these regions, autistic females had elevated rCVR compared to non-autistic females (rIFG: *β* = 0.19, p = 0.01; rOFC: *β* = 0.14, p = 0.009; Fig. 4). However, rCVR in autistic males was not significantly different compared to non-autistic males. In the rIFG, autistic females also had elevated rCVR compared to autistic males (*β* = 0.17, *p* = 0.03). In contrast, non-autistic females had lower rCVR compared to non-autistic males in both regions (rIFG: *β* = -0.08, p = 0.04; rOFC: *β* = -0.08, p = 0.01).

### 3.3 rCVR and functional connectivity

**Fig. 5.**
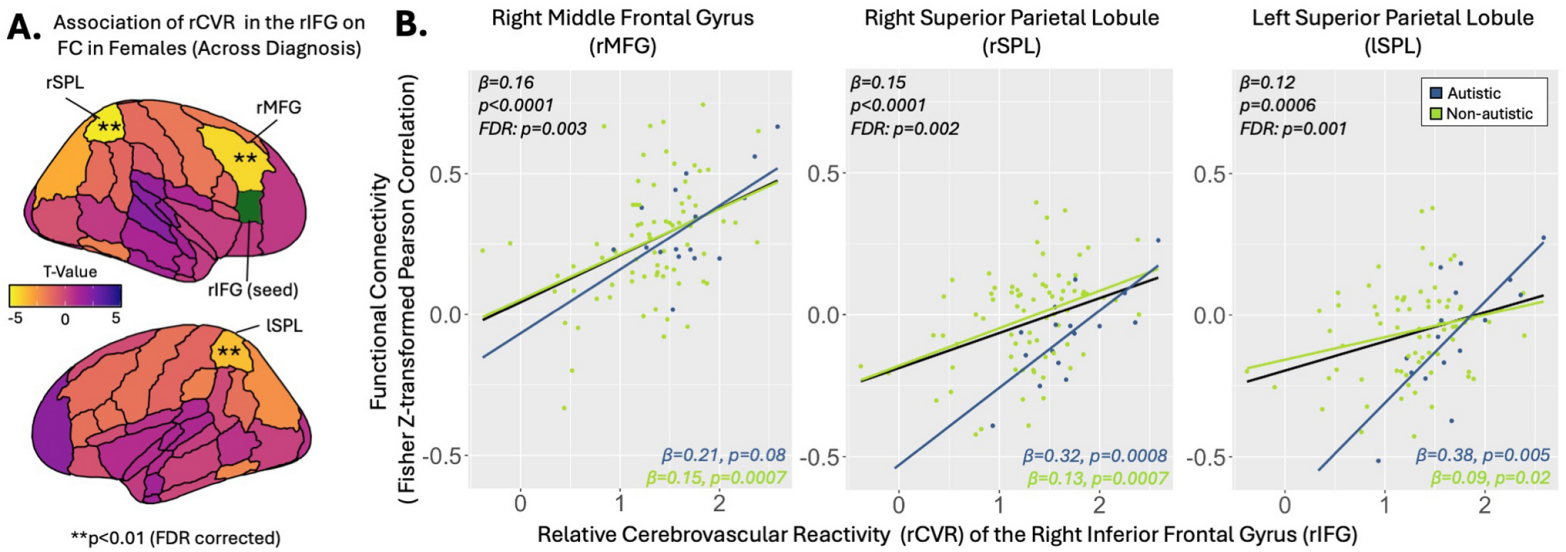
(A) Positive correlations between relative cerebrovascular reactivity (rCVR) in the right inferior front gyrus (rIFG) and functional connectivity in females. (B) rCVR in the rIFG was positively correlated with its functional connectivity to the right middle frontal gyrus (rMFG), right superior parietal lobule (rSPL), and left superior parietal lobule (lSPL) in females. All associations, except the association with FC to rMFG, remained significant in both the non-autistic female and autistic female subgroups. The association between rCVR in the rIFG and FC to the rMFG remained significant in non-autistic females, with a positive trend in autistic females.

We examined the association between rCVR and FC in regions with rCVR differences between diagnoses and/or sexes, as listed in Table 2. General linear models were performed within sex and diagnosis subgroups. We found that, within females, rCVR in the rIFG was positively associated with its FC to the rMFG (*β* = 0.16, p *< *0.0001, FDR p = 0.003), right superior parietal lobule (rSPL; *β* = 0.15, p *< *0.0001, FDR p = 0.002), and left superior parietal lobule (*β* = 0.12, p = 0.0006, FDR p = 0.001; Fig. 5). Within sex-by-diagnosis groups, these associations remained significant within non-autistic females in all three regions (rMFG: *β* = 0.15, p = 0.0007; rSPL: *β* = 0.13, p = 0.0007; lSPL: *β* = 0.09, p = 0.02) and within autistic females in the rSPL (*β* = 0.32, p = 0.0008) and lSPL (*β* = 0.38, p = 0.005; Fig. 5). RCVR in the rIFG was not significantly associated with FC to any region within male, autistic, or non-autistic subgroups (Table 3).

**Table 3.** General linear models of the association between relative cerebrovascular reactivity (rCVR) and functional connectivity.

		Females			Males			Autistic			Non-autistic		
Seed	FC region	Beta	p-value	FDR p-value	Beta	p-value	FDR p-value	Beta	p-value	FDR p-value	Beta	p-value	FDR p-value
Right inferior frontal gyrus	Right middle frontal gyrus	0.16	<0.0001	0.003*	0.05	0.08	0.55	0.07	0.05	0.86	0.09	0.003	0.12
	Right superior parietal lobule	0.15	<0.0001	0.002*	0.008	0.71	0.91	0.07	0.03	0.86	0.04	0.11	0.75
	Left superior parietal lobule	0.12	0.0006	0.001*	-0.02	0.46	0.89	0.05	0.11	0.86	0.01	0.60	0.92
Left posterior parahippocampal gyrus	Left supracalcarine cortex	0.14	0.0003	0.03*	0.01	0.60	0.88	0.03	0.38	0.79	0.05	0.05	0.32

* FDR p-value < 0.05.

Functional connectivity (FC); False discovery rate correction (FDR)

**Fig. 6.**
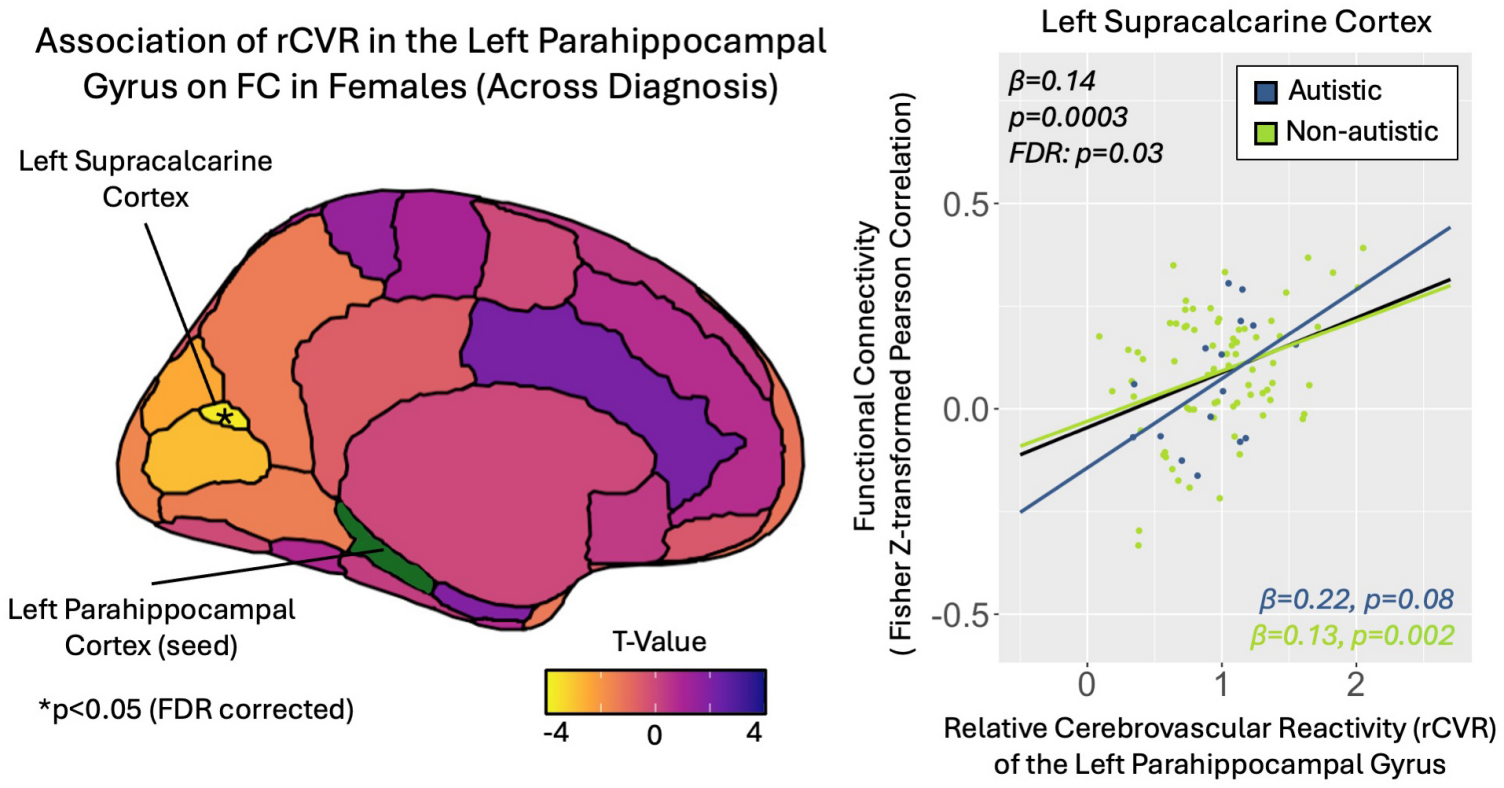
Positive correlations between relative cerebrovascular reactivity (rCVR) in the left parahippocampal gyrus (lPHC) and functional connectivity in females. rCVR of the lPHC was positively correlated with its functional connectivity to the left supracalcarine cortex. This association remained significant within the non-autistic female subgroup, with a positive trend in the autistic female subgroup.

We also identified a significant positive relationship between rCVR in the left posterior parahippocampal gyrus (lPHC) and its FC to the left supracalcarine cortex (lSCC) in females (*β* = 0.14, p = 0.0003, FDR p = 0.03; Fig. 6). Within sex-by-diagnosis groups, this association remained significant in non-autistic females (*β* = 0.13, p = 0.002; Fig. 6). RCVR in the lPHC was not significantly associated with FC to any region within male, autistic, or non-autistic subgroups (Table 3).

No significant associations were identified between rCVR in the rOFC, rFP, rMFG, rITG, subcallosal cortex, precuneus, or left planum polare and FC to any region within sex or diagnostic subgroups.

## 4 Discussion

For the first time, we leveraged rs-fMRI to evaluate regional CVR and its relationship to FC in autistic and non-autistic children, gaining insight into underlying cerebrovascular factors that may contribute to our interpretation of brain function differences in autism. We identified multiple differences in cerebrovascular function in autistic children in-vivo. Specifically, we observed sex-specific differences in the rIFG and rOFC, where autistic females had elevated rCVR compared to non-autistic females, but males did not show the same diagnostic difference. In these regions, sex differences also contrasted by diagnostic group; females had lower rCVR compared to males in the non-autistic group, but higher rCVR compared to males in the autistic group. Across sex, autistic children showed higher rCVR in the rFP and rMFG, but lower rCVR in the rITG compared to non-autistic children. Using the same rs-fMRI data, we measured the FC of these regions across the brain and found significant associations between rCVR and FC, specifically in the rIFG and lPHC. In particular, we observed strong positive associations only within females between: (1) rCVR in the rIFG and its FC to the rMFG, rSPL, and lSPL, and (2) rCVR in the lPHC and its FC to the lSCC, indicating potential regional connections and subgroups where FC may be more influenced by CVR in children. Significant associations between rCVR and FC were observed in regions with sex-by-diagnosis differences in rCVR (i.e., rIFG), in addition to regions with overall sex differences in rCVR (i.e., lPHC). Together, our study highlights functional cerebrovascular differences in autism, including sex-specific differences, that provide a more comprehensive view of the neurobiology of autism and improve interpretations of FC analyses in children.

Our findings of rCVR differences in autism both within and across sex add in-vivo human evidence to support recent preclinical and post-mortem studies that observed cerebrovascular differences in autism (Azmitia et al., 2016; Ouellette et al., 2020). CVR is widely used to study cerebrovascular conditions, such as cerebral steno-occlusive diseases (Donahue et al., 2013; Leoni et al., 2012), small vessel disease (Blair et al., 2016; Sleight et al., 2023), and stroke (Geranmayeh et al., 2015; Krainik et al., 2005), in which differences in CVR are attributed to aberrant function of cerebrovascular endothelial cells and/or smooth muscle cells surrounding cerebrovasculature. These biological interpretations of atypical CVR derived from fMRI are consistent with mechanisms of cerebrovascular endothelial cell dysfunction identified in preclinical and post-mortem studies of autism (Azmitia et al., 2016; Fiorentino et al., 2016; Jiménez et al., 2022; Ouellette et al., 2020). A previous study in a mouse model of syndromic autism also identified sex-specific diagnostic differences in vascular reactivity; however, they identified decreased vascular reactivity in male mice with a 16p11.2 deletion, specifically in response to pharmacological vasodilators in the middle cerebral artery (Ouellette et al., 2020). These inconsistent findings could be due to differences in sample (e.g., preclinical vs. clinical, 16p11.2 deletion syndrome vs. autism), methodology (e.g., pharmacological vasodilators vs. intrinsic CO_2_, microscopy vs. fMRI), age range, and cerebral vessels of interest. Nevertheless, both studies highlight that potential vascular differences in autism may be sex-specific.

Although future work relating direct measures of vasculature to neuroimaging metrics are needed to confirm these biological interpretations, our findings indicate group-level differences in the local BOLD response to systemic physiology that are consistent with underlying changes in cerebrovascular function and regulation. The regions identified in this study can guide future research into how vascular conditions that co-occur with autism, as indicated by recent studies that found an increased risk of cardiovascular conditions and stroke in autistic individuals (Dhanasekara et al., 2023; Jin et al., 2024), may impact vascular function in the brain. Increased CVR, though generally associated with better cerebrovascular health in aging, may have different effects on neurovascular coupling during development. Further studies investigating CVR in children are needed to understand how the relationship between CVR and cognition may change across the lifespan.

Use of rs-fMRI enabled us to simultaneously investigate regional differences in rCVR and identify potential functional networks that are vulnerable to cerebrovascular differences in autism. Recent studies provide evidence for “cerebrovascular networks” that spatially parallel canonical neuronal networks and exhibit distinct cerebrovascular physiology (Bright et al., 2020; J. E. Chen et al., 2020). Thus, atypical cerebrovascular function could spatially coincide with and contribute to aberrant neuronal network connectivity. In our study, autistic girls exhibited elevated rCVR, specifically in right-frontal gray matter regions that have been implicated in the ventral attention network (VAN) (Cazzoli et al., 2021; Trambaiolli et al., 2022). The VAN is a right-lateralized functional network that is activated when orienting attention to relevant environmental stimuli and has been highly implicated in autism (Corbetta et al., 2008; Corbetta & Shulman, 2002; Farrant & Uddin, 2016). Attentional differences are consistently reported in autism, with autistic children often exhibiting early differences in attention tasks, including visual orienting and auditory detection paradigms (Elsabbagh et al., 2009; Zwaigenbaum et al., 2005). Both hyper- and hypo-connectivity have been identified within the VAN and salience networks in autistic children, alluding to inconsistent alterations in neuronal circuitry in the literature (Abbott et al., 2016; Farrant & Uddin, 2016; Green et al., 2016; Lawrence et al., 2020). Differences in the underlying “cerebrovascular network” could contribute to or precede differences in FC. While previous studies have investigated the influence of CVR on rs-fMRI metrics in adults and identified regional differences in these effects (D. Y. Chen, Di, Yu, et al., 2024; Chu et al., 2018; Golestani et al., 2016), relationships between CVR and FC have yet to be examined in children and neurodevelopmental conditions. In our study, we found that rCVR, specifically in the rIFG and lPHC, was strongly associated with FC in girls. Both short range (e.g. rIFG-rMFG) and long range (e.g. rIFG-lSPL) functional connections that are associated with inhibition and attentional control were positively correlated with rCVR (Hampshire et al., 2010; Japee et al., 2015), suggesting that cerebrovascular differences (i.e., rCVR) may influence FC measures across the brain in children. Furthermore, relationships between rCVR and FC were only identified within females, indicating that the effect of rCVR on FC may differ between sexes in this age group. These interactions should be further explored to improve FC interpretations in pediatric and autistic populations.

Interestingly, in the rIFG and rOFC, increased rCVR was found in autistic females, but not autistic males. This is consistent with known sex differences in cerebrovascular function related to sex hormone regulation of vascular dynamics (Robison et al., 2019; Sabra et al., 2021). A previous study using 4D-flow MRI to measure absolute CVR during hypercapnia (i.e., breathing elevated CO_2_) identified differences absolute CVR in young adult females compared to males in the internal carotid and MCA (Miller et al., 2019). While previous CVR studies in children lack sufficient sample size to examine sex differences (D. Y. Chen, Di, & Biswal, 2024; Leung et al., 2016; Zvolanek et al., 2022), studies of cerebral blood flow (CBF) during typical development, which correlates with CVR in adolescence (Zvolanek et al., 2024), have identified sex differences in CBF that emerge after puberty. Satterthwaite et al. (2014) identified that females showed greater CBF than males only after the pubertal stage, in regions such as the right dorsolateral prefrontal cortex, right insula, and ventromedial prefrontal cortex. Baller et al. (2022) also found increased neurovascular coupling in females compared to males during puberty, including in the bilateral dorsolateral prefrontal cortex and medial frontal cortex. In parallel, studies of adolescence in autism indicate that autistic girls exhibit an earlier onset of puberty relative to non-autistic females and autistic males (Corbett et al., 2020). Together, our finding of elevated rCVR in autistic females compared to non-autistic females and autistic males during middle childhood, on the cusp of adolescence, may be related to an earlier onset of puberty and elevated CBF in frontal regions in females after puberty. However, our finding of contrasting sex differences between autistic and non-autistic groups suggests that puberty-related changes in CVR do not fully explain the diagnostic differences we observed in females. Further studies will evaluate these rCVR differences longitudinally, pre- and post-puberty, in autistic children to disentangle sex-specific cerebrovascular differences.

Autism is not a primarily cerebrovascular condition, which may contribute to why none of the rCVR differences pass FDR correction when considering 91 gray matter regions of the Harvard-Oxford atlas. We took an exploratory approach to comprehensively characterize our novel metric across all gray matter in autism and fill a fundamental gap in the literature. Our findings are the first to identify rCVR differences in autism in-vivo that can guide future analyses in cerebrovascular differences in autism that are largely understudied. Subsequent FC analyses based on regions identified in these exploratory analyses showed strong associations that passed multiple comparisons correction. Furthermore, our main findings were identified in autistic females, who are largely underrepresented in neuroimaging research studies, and can contribute to further studies that disentangle the etiology in autism in females at multiple levels of neurobiology where there are also known sex differences in non-autistic populations.

Moreover, we approximate arterial CO_2_ changes from the frequency-filtered global BOLD signal, based on known respiratory dynamics at 0.02–0.04 Hz. This resting-state method has been previously validated with gold-standard CVR measures using gas challenges and respiratory CO_2_ recordings and proven to be highly reproducible across sessions within participants (Liu et al., 2017). However, other physiological dynamics such as cardiac or autonomic changes that can be dependent on behavior, arousal, or systemic regulation (Bolt et al., 2025), in addition to CO_2_ changes, contribute to the global BOLD signal at 0.02–0.04 Hz (Q. N. Lee et al., 2023) and may differ in some participants with autism (Cheng et al., 2020). Furthermore, our estimates of CO_2_ fluctuations, using the global BOLD signal, are relative, which precludes us from drawing conclusions on quantitative, absolute CVR. Lastly, without inducing large fluctuations in CO_2_ through gas challenges or breathing paradigms, our CVR measure has a lower signal-to-noise ratio and sensitivity to detect differences between groups.

Despite these limitations, we capitalized on the advantages of rs-fMRI derived rCVR to identify sex-specific, regional differences in rCVR in autistic children and relate them to FC. For example, since rCVR is based on standard rs-fMRI scans that are more accessible to autistic and non-autistic children, we could utilize large repositories and significantly increase our sample size and statistical power. Furthermore, due to within subject normalization, our relative CVR measurements are less susceptible to motion confounds (Fig. 2), which are critical to account for in neuroimaging investigations of autistic and non-autistic children. Despite the study limitations, our findings provide novel insight into cerebrovascular differences in autism and how they contribute to FC that could not be otherwise examined in autistic children in-vivo.

Although the ABIDE dataset allows for investigation of a large sample size and participant diversity, there are several limitations to consider. First, since ABIDE is a multi-site database, we implemented rigorous data harmonization and site exclusion criteria, which tempered our achievable sample size. There is a large sample size difference between subgroups and our main results of sex-specific effects in autism are derived from a relatively small sub-sample of 16 autistic females. Thus, our findings in autistic females are more susceptible to outlying datapoints and should be interpreted with caution. Furthermore, ABIDE is cross-sectional with inconsistent clinical and behavioral information, thus we are unable to explore links between our imaging findings and attentional skills in this cohort. In future studies, we will examine rCVR differences longitudinally, from early childhood to post-adolescence, with additional behavioral and pubertal information. These studies will allow us to identify critical periods where rCVR differences emerge, disentangle effects of puberty, and extend our findings to behavior.

## 5 Conclusions

In summary, we leveraged resting-state rCVR mapping and the accessibility of resting-state fMRI to identify cerebrovascular contributions to autism and their relationship to FC findings in pediatric and autistic populations. For the first time, we identified sex-specific differences in CVR in autism, particularly in right-frontal brain regions associated with attentional control. Furthermore, we demonstrated that resting-state rCVR was positively correlated with FC, specifically in functional connections of the rIFG and lPHC in girls, suggesting that cerebrovascular effects on FC may differ regionally and between sexes in children. Our findings provide novel neurobiological insight into the etiology of autism and highlight potential cerebrovascular differences that can inform our interpretations of FC in pediatric and autistic groups.

## Supplementary Material

Supplementary Material

## Data Availability

The dataset analyzed is available in the ABIDE repository (https://fcon_1000.projects.nitrc.org/indi/abide/). All the analyzed data and software source code supporting conclusions presented in this work are available from the corresponding author upon email request.
