# Supplementary material for: Sex-specific cerebrovascular reactivity differences in autistic children related to functional connectivity
Source: Imaging Neurosci (Camb). 2025 Nov 17;3:IMAG.a.1022. doi: 10.1162/IMAG.a.1022 (PMC12624364; doi:10.1162/IMAG.a.1022)
Supplement: Supplementary Material [file IMAG.a.1022_supp.pdf]

Supplementary Materials

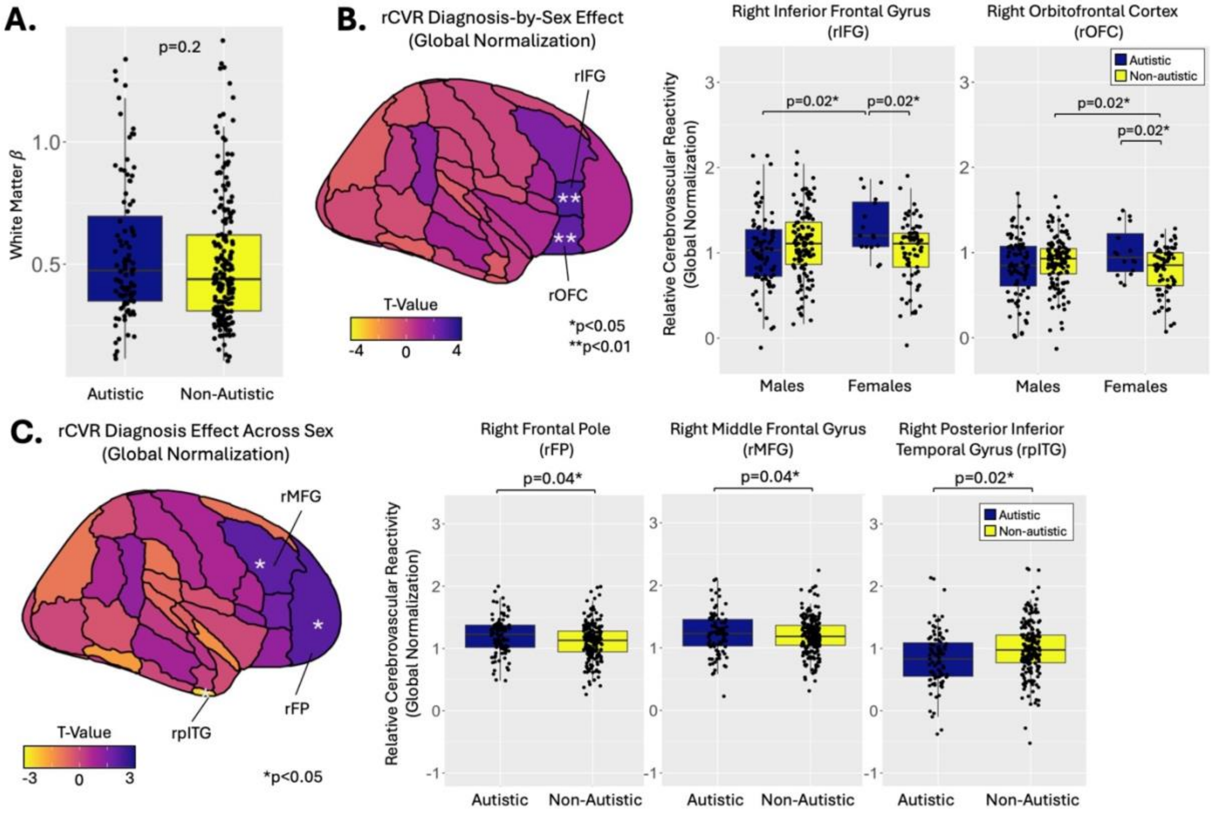

Supplementary Figure 1. White Matter Normalization. (A) White matter *β* did not significantly differ between autistic and non-autistic groups. **(B-C)** Comparisons of relative cerebrovascular reactivity (rCVR), normalized to global *β,* yielded the same results found in our analysis using rCVR normalized to white matter *β.*

Supplementary Table 1 Site Characteristics

	Resting-state functional MRI Acquisition					Number of Participants (n)				
**Site ID**	**Scanner**	**TR (ms)**	**TE (ms)**	**Resting-state Scan Duration (mm:ss)**	**Resting-state Voxel size (mm)**	**Total**	**Autistic Males**	**Autistic Females**	**Non-Autistic Males**	**Non-Autistic Females**
**ABIDE I**										
STANFORD	GE Signa	2000	30	6:00	3.1x3.1x4.5	17	8	2	5	2
UCLA_1	Siemens Trio	3000	28	6:06	3.0x3.0x4.0	15	7	2	5	1
UM_1	GE Signa	2000	30	10:00	3.4x3.4x3.0	21	5	1	12	3
YALE	Siemens Trio	2000	25	6:40	3.4x3.4x4.0	12	5	1	6	0
**ABIDE II**										
ABIDEII-GU_1	Siemens Trio	2000	30	5:14	3.0x3.0x3.0	34	15	1	10	8
ABIDEII-KKI_1	Philips Achieva	2500	30	6:40	2.7x2.7x3.0	122	15	4	61	42
ABIDEII-OHSU_1	Siemens Trio	2500	30	5:07	3.8x3.8x3.8	54	15	3	19	17
ABIDEII-SDSU_1	GE MR750	2000	30	6:10	3.4x3.4x3.4	19	9	2	7	1

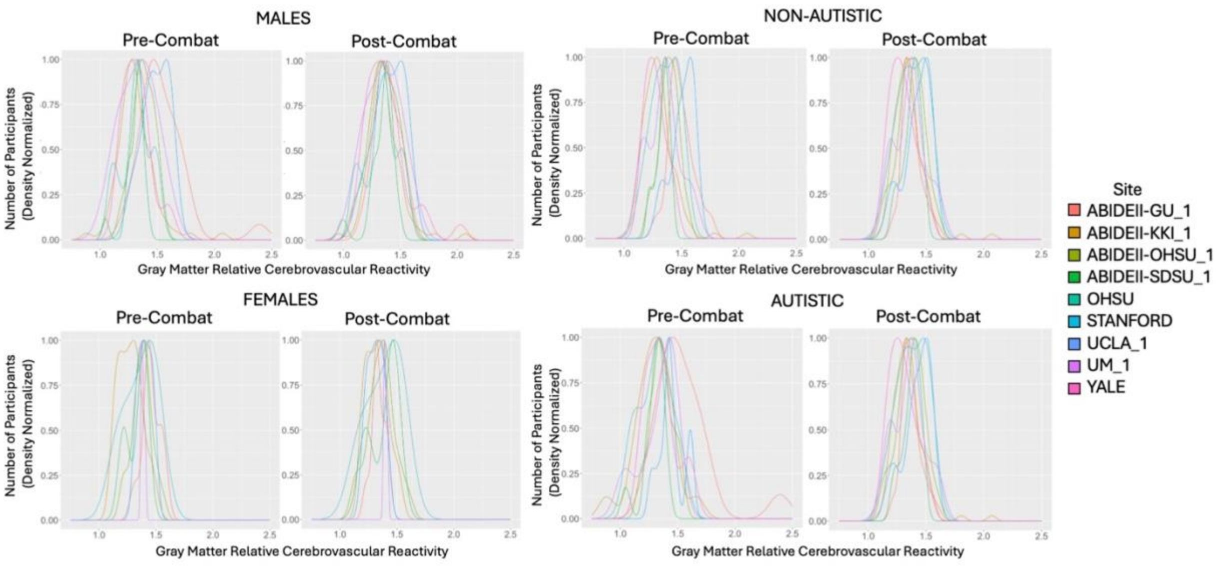

Supplementary Figure 2. Gray matter relative cerebrovascular reactivity (rCVR) values pre- and post- ComBat data harmonization. Features of the ComBat model represented regional gray matter averages, delineated using the Harvard-Oxford atlas. Diagnosis, sex, and mean-centered age were included as biological covariates of interest. Distributions reflect CVR of global gray matter compared to white matter.

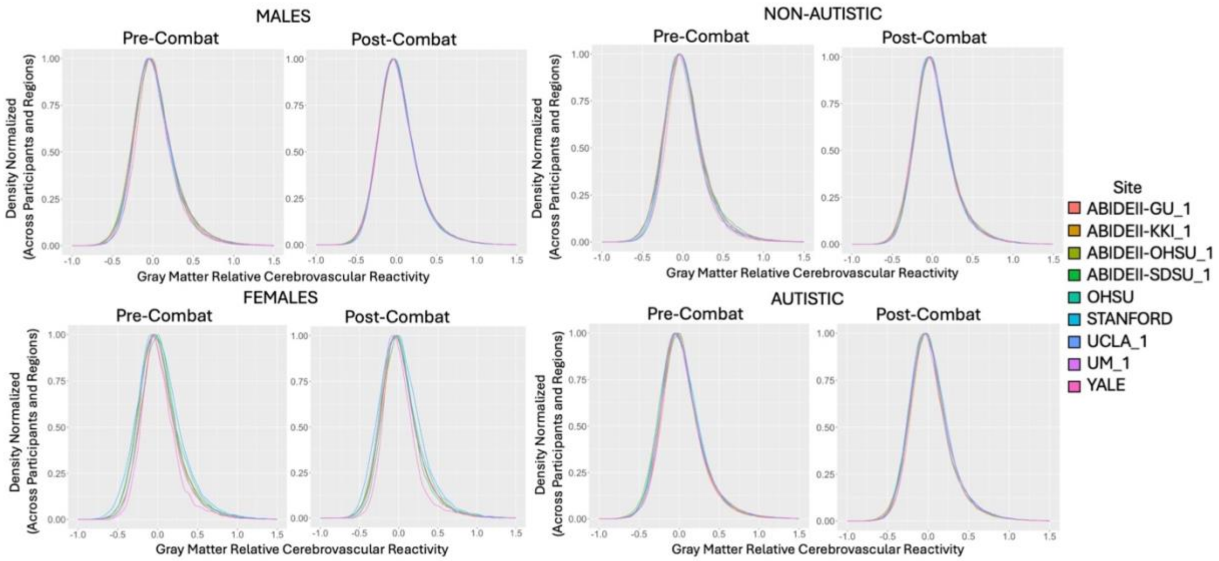

Supplementary Figure 3. Functional connectivity values pre- and post- ComBat data harmonization. Features of the ComBat model represented region-to-region functional connectivity values for all combinations of gray matter regions in the Harvard-Oxford atlas. Diagnosis, sex, and mean-centered age were included as biological covariates of interest. Distributions reflect rCVR across all participants and gray matter regions.
